# Brain Volume Measures in Adults with MOG-Antibody-Associated Disease: A Longitudinal Multicenter Study

**DOI:** 10.3390/jcm14072445

**Published:** 2025-04-03

**Authors:** Riccardo Orlandi, Sara Mariotto, Francesca Gobbin, Francesca Rossi, Valentina Camera, Massimiliano Calabrese, Francesca Calabria, Alberto Gajofatto

**Affiliations:** 1 Department of Neuroscience, Biomedicine and Movement Sciences, University of Verona, Piazzale L.A. Scuro 10, 37134 Verona, Italy; sara.mariotto@aovr.veneto.it (S.M.); francesca.gobbin@gmail.com (F.G.); valentina.camera@univr.it (V.C.); massimiliano.calabrese@univr.it (M.C.); alberto.gajofatto@univr.it (A.G.); 2 Neurology Unit, Mater Salutis Hospital, 37045 Legnago, Italy; francesca.rossi@aulss9.veneto.it; 3 Neurology Unit, Ospedale Borgo Trento, Azienda Ospedaliera Universitaria Integrata, 37126 Verona, Italy; francesca.calabria@aovr.veneto.it

**Keywords:** MOGAD, multiple sclerosis, MRI, brain atrophy, longitudinal study

## Abstract

**Background/Objectives**: Little is known about the impact of myelin oligodendrocyte glycoprotein antibody-associated disease (MOGAD) on brain atrophy. This multicenter longitudinal study compares brain MRI volumes and T2 lesion volume between MOGAD patients, relapsing-remitting MS (RRMS) patients and a healthy control (HC) group with brain MRI scans available from an online repository. **Methods**: In total, 16 adult MOGAD patients (9 F) were age- and sex-matched with 44 RRMS patients (17 F) recruited in Verona MS Center and 14 HC subjects. The availability of two brain MRI scans performed 18 ± 6 months apart was mandatory for each patient. Annual percentage brain volume change (PBVC/y), baseline global brain, white matter (WM), gray matter (GM) regional brain volumes and T2 lesion volume were compared between groups. **Results**: PBVC/y was lower in MOGAD than in RRMS patients (*p* = 0.014) and lower in HC subjects than in MS patients (*p* = 0.005). Overall, MOGAD showed higher mean global brain (*p* = 0.012) and WM volume (*p* = 0.024) but lower median T2 lesion volume at timepoint 1 (*p* < 0.001); T2 lesion volume increased over time in the RRMS (*p* < 0.001) but not in the MOGAD cohort (*p* = 0.262). **Conclusions**: The structural brain MRI features of MOGAD show higher global brain and WM volumes and lower brain volume loss over time compared to RRMS, suggesting different underlining pathogenetic mechanisms.

## 1. Introduction

Myelin oligodendrocyte glycoprotein (MOG) antibody-associated disease (MOGAD) is a recently recognized demyelinating disease of the central nervous system (CNS) with a broad clinical spectrum, which includes optic neuritis (ON), transverse myelitis (TM), brainstem syndrome, acute disseminated encephalomyelitis (ADEM) [1], as well as encephalitis and seizures [2].

The clinical features of MOGAD partly overlap with multiple sclerosis (MS) and aquaporin-4 antibody (AQP4-Ab) seropositive neuromyelitis optica spectrum disorders (NMOSD-AQP4); however, different immunopathogenetic mechanisms underlie the three diseases [3]. MOGAD is an immune-mediated disease in which a direct pathogenetic role of MOG-IgG along with the activation of T-cell-mediated inflammation has been postulated, causing oligodendrocyte and myelin damage. MS shows immune-mediated demyelination as well, with an interplay of innate and adaptive immunity orchestrating a response against autoantigens that are still to be identified. Conversely, NMOSD-AQP4 is an astrocytopathy in which AQP4-Abs play the primary pathogenetic role.

It is important to find the clinical and paraclinical features that distinguish MOGAD from NMOSD-AQP4 and MS, as the three diseases have different evolution and treatment strategies [4,5]. So far, magnetic resonance imaging (MRI) has proven to be a helpful tool in achieving an early differentiation of these conditions, and many studies have described the distribution and morphology of demyelinating lesions associated with MOGAD [6]. Brain MRI in MOGAD can be normal in up to 50% of patients; lesions are often large, poorly demarcated and extend from the cortical–subcortical region to the white matter and the basal ganglia. Infratentorial and brainstem lesions are also present and tend to localize to the midbrain, the olivary bodies and the cerebellar peduncles. Gadolinium-enhancing (Gd+) lesions in MOGAD are often described as nodular or perivascular within white matter lesions rather than the “open ring” pattern typically observed in MS [7].

In MS, white matter lesions tend to affect specific brain regions, such as the periventricular and juxtacortical white matter, the corpus callosum and the infratentorial areas; in NMOSD-AQP4, brain lesions are mostly located in areas with high AQP4 expression (e.g., periependymal lesions surrounding the ventricles or involving corticospinal tracts) [8].

Little is known about the presence of whole and regional brain atrophy in MOGAD patients, measured using a volumetric MRI approach, while in MS—both in the relapsing-remitting (RRMS) and in the progressive phenotypes—there is strong evidence of brain volume loss during the course of the disease [9]. The comparison of the extent of brain atrophy in MOGAD and MS patients could give us clues as to the neuropathological substrates of these two diseases, as well as to the different treatment strategies to adopt.

Therefore, the primary endpoint of this multicenter study is to compare the degree of global brain atrophy, expressed as annualized percentage brain volume change (PBVC/year), in two matched cohorts of patients affected by MOGAD or MS and in a group of HC subjects. The PBVC/year is assessed through longitudinal analysis of two MRI scans performed at two different timepoints. The secondary objectives of the study include a cross-sectional analysis of brain lesion distribution and pattern in MOGAD and MS patients, a comparison of baseline values of global brain, white matter (WM), gray matter (GM) and regional brain volumes between the two cohorts and a comparison of differences in T2 lesion load between the two groups; additionally, a longitudinal assessment of T2 lesion load progression between the two cohorts is performed.

## 2. Materials and Methods

### 2.1. Patient Enrollment

This longitudinal multicenter observational study involved three centers in Italy (Ospedale Borgo Roma and Ospedale Borgo Trento (Verona University Hospitals) and Mater Salutis Hospital, Legnago). MOGAD patients were prospectively recruited at each participating center. Enrollment started on 31 January 2017 and ended on 30 June 2022. The study was approved by the ethics committee for clinical research of Verona and Rovigo provinces, Italy (n. 1052CESC). Eligible cases were recruited according to the following inclusion criteria: patients ≥ 18 years old of both sexes; signed written informed consent form; at least one clinical episode compatible with the initial demyelinating event (IDE) of the CNS; MOG-IgG positivity on serum and/or cerebrospinal fluid (CSF) sample; clinical follow-up of at least six months. After enrollment, a screening assessing the quality of baseline brain MRI scan was performed by an investigator at Verona MS Center to evaluate whether all scans could undergo processing with dedicated software. Patients whose MRI scans did not qualify for further analysis were excluded from the study.

MS patients were recruited at Verona MS Center according to the following inclusion criteria: patients ≥ 18 years old, age- (±5 years) and sex-matched with MOGAD cases; MS diagnosis according to the 2017 McDonald Criteria; signed written informed consent form.

For the longitudinal and cross-sectional analysis of PBVC/y, global brain, WM and GM volumes, a comparison of MOGAD and MS patients with a group of age- and sex-matched control subjects (all Caucasians) was performed using brain MRI scans performed 2 years apart available from an online repository (“Wayne State Study 11 Dataset”, available at http://fcon_1000.projects.nitrc.org/indi/retro/wayne_11.html, accessed on 20 June 2024).

### 2.2. Study Design

**Figure 1:**
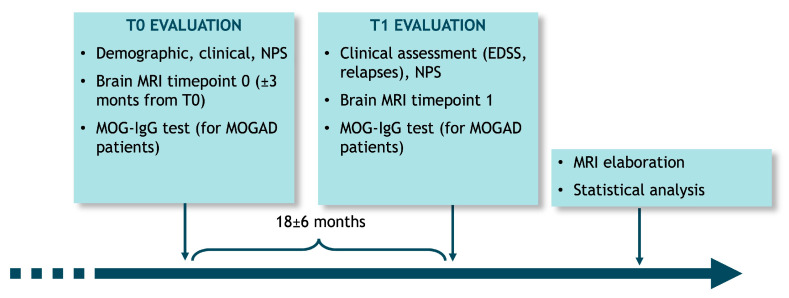
Study design.

After signing written informed consent forms, patients entered the study at baseline (T0), where demographic and clinical variables were collected. A neuropsychological assessment was also performed, as well as a brain MRI scan (±3 months from baseline) and a serum MOG-IgG test. At T1 evaluation, which followed the baseline visit after 18 ± 6 months, a clinical assessment was performed, as well as a new brain MRI and a serum MOG-IgG test for MOGAD patients. Each patient underwent a longitudinal and cross-sectional (at baseline) evaluation of brain MRI images through dedicated software (Figure 1).

### 2.3. Clinical Assessment

The clinical variables collected at baseline and at T1 included demographic data (sex, age, ethnicity), clinical symptoms and the anatomical area involved at ADS onset, disease duration at the second MRI timepoint, Expanded Disability Status Scale (EDSS) score at the two timepoints, the interval between the two MRI exams, previous relapses before the first MRI and during the follow-up period. These data were entered in a dedicated database.

### 2.4. MRI Data Protocol

MRI scans were acquired at each participating center using the same MRI protocol and scanner for both timepoints. T1-weighted 3D scans (on 1.5 Tesla magnets—Philips Ingenia^®^ (Philips Healthcare, Eindhoven, The Netherlands) and GE Signa^®^ (GE Healthcare, Milwaukee, WI, USA)) were used for the estimation of PBVC/y and for the cross-sectional assessment of whole brain, GM, WM and regional brain volumes.

The 3D FLAIR scans were used for T2 lesion distribution and volume assessment. Double inversion recovery (DIR) 3D sequences were used for the assessment of cortical lesions, whenever available. The evaluation of the number, distribution and characteristics of brain lesions at baseline was performed visually at each participating center by the local investigator, according to a pre-specified data log made available to the centers at the beginning of the study.

Whole brain volume changes between the two timepoints were assessed using SIENA software, version 6.0.7 [10], part of the FMRIB Software Library (FSL—www.fmrib.ox.ac.uk/fsl/, accessed on 10 February 2022). This registration-based method uses images from two timepoints to assess brain volume changes by giving an estimate of percentage brain volume change (PBVC) between the two scans. The PBVC value for each patient/control subject was subsequently annualized (PBVC/y). Whole brain, white matter (WM) and gray matter (GM) volumes at baseline were assessed using SIENAX software, version 6.0.7 [11], part of the same FMRIB suite described earlier. SIENAX measures the volume of the brain from a single MRI scan and then normalizes it to a standard skull to yield a normalized brain volume (NBV). NBV can be thought of as the fraction of the skull that is filled with brain. It also provides normalized white and gray matter volume values.

T2 lesion volume (T2LV) was assessed for each timepoint using ITK-SNAP [12] (http://www.itksnap.org/pmwiki/pmwiki.php, accessed on 5 June 2021), a semi-automated software that allows lesion segmentation on 3D FLAIR sequences. The T2 lesion mask automatically generated through ITK-SNAP was then visually inspected and manually corrected when necessary by an investigator from Verona MS Center.

Regional volumes of cerebrum, cerebellum, thalamus, putamen, caudate and hippocampus were measured using VolBrain (version 1.0), a recently published automated open-source software. It uses anonymized T1-weighted 3D images in .nifti format for elaboration and generates a report with volumes of major intracranial tissues [13].

All analyses were carried out by an assessor from Verona University Hospital who was unblinded to patients’ diagnoses.

### 2.5. MOG-IgG Testing

MOG-IgG titers were assessed at the Verona Neuropathology Laboratory using an anti-total-IgG recombinant live-cell-based immunofluorescence assay with HEK293A cells transfected with full-length MOG. Live cell assays are currently considered the gold standard for detection of circulating antibodies [14], although their limitation is the need for an in-house setup, which is difficult to standardize. We assessed the MOG-IgG titer in both serum and CSF, when available, according to recent evidence of CSF MOG-IgG in seronegative patients with a clinical phenotype highly compatible with MOGAD [15]. For serum MOG-IgG detection, the assay was performed at 1:20 and 1:40 dilutions, and MOG-IgG positivity was titrated with serial dilutions; MOG-IgG positivity was set at a titer of ≥1:160, as previously described [16,17,18]. For the detection of CSF MOG-IgG assay, samples were tested undiluted and at 1:2 dilution, with subsequent serial titrations [15].

### 2.6. Statistical Analysis

Given the lack of literature on the expected brain volume change in MOGAD patients, the sample size calculation for defining the significance in annual PBVC differences between MOGAD and MS subjects was based on studies comparing MS patients with healthy individuals. Specifically, it was assumed that the expected PBVC value for MOGAD subjects could be comparable to that of RRMS patients (null hypothesis), with an annual percentage change of −0.52 ± 0.29% [19]. Based on this assumption, a sample size of 20 MOGAD patients and 30 MS patients was calculated to achieve 80% statistical power with 95% confidence interval. The target sample size for MOGAD subjects was increased to 25 to mitigate the impact of potential dropouts on study power.

Quantitative variables were expressed as mean and standard deviation or, in case of a non-parametric distribution, as median and range, or both. Categorical variables were expressed as absolute and relative frequencies. To evaluate the normal distribution of the variables, we performed a Kolmogorov–Smirnov test, and we visually inspected the frequency histograms. For normally distributed variables, an independent sample *t*-test was applied to assess the mean differences between MOGAD and MS; a comparison between continuous variables between MOGAD, MS and HC subjects was computed using one-way analysis of variance (ANOVA), followed by post hoc Tukey’s multiple comparison test. For non-normally distributed variables, a Kruskal–Wallis test was performed, as appropriate. For categorical variables, a chi-squared test was performed to assess the differences between groups. Statistical significance was set at a two-sided *p* value of <0.05. Statistical analysis was conducted using Jamovi software (Version 2.5; The Jamovi Project, available at https://www.jamovi.org, accessed on 25 July 2024).

### 2.7. Patient and Public Statement

Neither the patients nor the public were involved in the design, conduct, reporting or dissemination plans of our research.

## 3. Results

### 3.1. Demographic and Clinical Features

The study recruited 16 MOGAD adult patients (9 F); the MS cohort consisted of 44 patients with RRMS (26 F). Brain MRI scans from 14 age- and sex-matched control subjects were also analyzed for comparison with MOGAD and MS patients.

The demographic, clinical and paraclinical characteristics of MOGAD, MS and HC subjects are summarized in Table 1.

### 3.2. Brain MRI Lesion Characteristics

**Figure 2:**
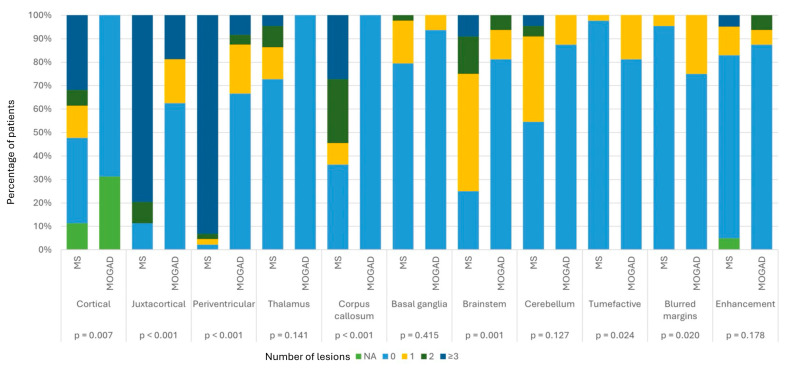
Percentage of MOGAD versus MS patients with 0, 1, 2 or ≥3 lesions in different brain areas.

The distribution and the number of brain MRI lesions are summarized in Figure 2.

Eight MOGAD patients (50%) did not show brain lesions. MOGAD patients had significantly fewer cortical (*p* = 0.007), periventricular, juxtacortical and callosal (*p* < 0.001) lesions compared with the MS cohort; on the contrary, we found a significantly higher number of tumefactive lesions (*p* = 0.024) and lesions with blurred margins (*p* = 0.02) in MOGAD patients than in MS subjects.

### 3.3. Brain Volume Measures

**Figure 3:**
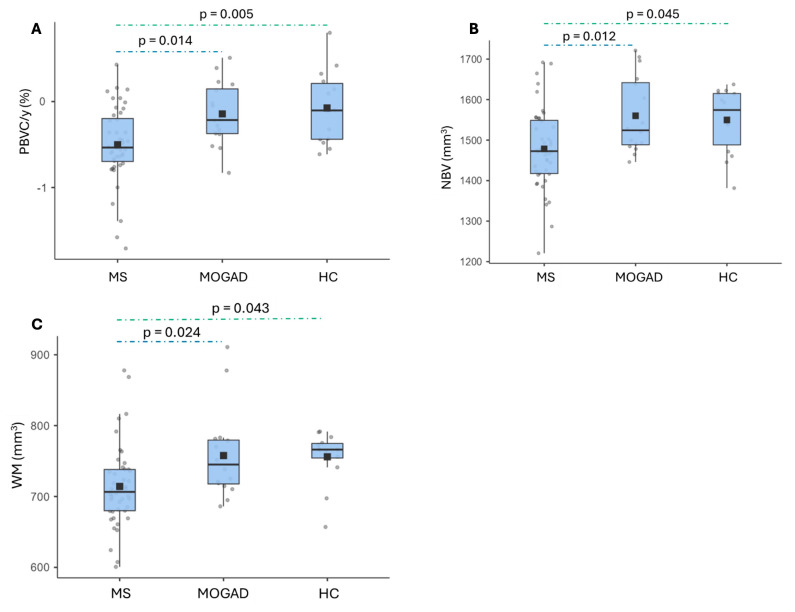
Brain volume measures on MRI scans. Horizontal black bars represent median values. Squares are means. (**A**): Annualized percentage brain volume change in MS, MOGAD and HC subjects; (**B**): NBV distribution in MS, MOGAD and HC subjects (cm^3^); (**C**): WM volume distribution in MS and MOG patients.

Mean PBVC/y was significantly different among MOGAD patients (−0.143 ± 0.366%), MS patients (−0.502 ± 0.444) and HC subjects (−0.07 ± 0.425%, *p* = 0.002); a post hoc analysis using Tukey’s test showed significantly higher mean PBVC in MS patients compared to MOGAD patients (mean difference −0.359, *p* = 0.014) and to HC subjects (mean difference −0.428, *p* = 0.005) (Figure 3A).

The cross-sectional analysis performed with SIENAX showed that the mean NBV was significantly different among MS (1478,37 ± 99.95 cm^3^), MOGAD (1560.1 ± 93.96 cm^3^) and HC subjects (1549.75 ± 80.58 cm^3^, *p* 0.007). The post hoc analysis revealed a significant difference between MS and MOGAD cohorts (mean difference −81.73 cm^3^, *p* = 0.012), and a borderline significant difference was noted between MS and HC groups (mean difference −71.4 cm^3^, *p* = 0.045) (Figure 3B).

The mean GM volume did not differ significantly among the groups. The mean WM volume was significantly different between MS (714.16 ± 57.75 cm^3^), MOGAD (757.72 ± 61.71 cm^3^) and HC subjects (755.99 ± 36.98 cm^3^, *p* = 0.006). The post hoc analysis showed a significant difference between MS and MOGAD cases (mean difference −43.6 cm^3^, *p* = 0.024) and a borderline significant difference between MS and HC cohorts (mean difference −41.83 cm^3^, *p* = 0.043) (Figure 3C).

No significant differences were observed for the median regional volumes of cerebellum, thalamus, caudate, putamen and hippocampus.

**Figure 4:**
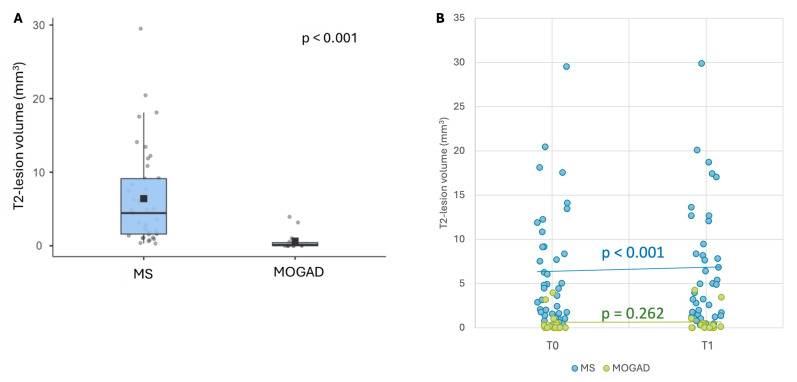
T2 lesion load. (**A**): T2 lesion volume at T0. Horizontal black bars represent median values. Squares are means; (**B**): T2 lesion volume distribution at T0 and T1 in MOGAD and MS patients.

At baseline, the median T2 lesion volume was significantly higher in MS (4.46 cm^3^, range 0.31–94.3) compared to MOGAD patients (0.14 cm^3^, range 0–105) (Figure 4A). The median T2 lesion volume increased from T0 (4.46 cm^3^, range 0.31–94.3) to T1 (4.96 cm^3^, range 0.3–95.1, *p* < 0.001) in MS cases but not in MOGAD patients (T0: 0.14 cm^3^, range 0–105; T1: 0.125 cm^3^, range 0–105, *p* = 0.262) (Figure 4B).

## 4. Discussion

### 4.1. Longitudinal Analysis of Brain Volumes

In our study, the mean PBVC/y was different between MOGAD, MS and HC subjects, and it was particularly significantly lower in MOGAD patients compared to MS patients. The mean PBVC/y observed in the MS group was similar to the estimates available from previous studies [19,20]. These results are partly consistent with a previous preliminary analysis [21] from our group, where it was observed that the mean PBVC/y was lower in MOGAD patients than in RRMS patients in a subgroup of patients aged <60 years and with a disease duration of ≤10 years, with borderline significance. However, this study was limited by large heterogeneity in the MRI analysis, which encouraged us to replicate these results in a study with fewer methodological issues and with a more consistent sample. There are very little data regarding the mean PBVC/y in MOGAD patients. In particular, a recent study among 8 MOGAD, 22 NMOSD-AQP and 34 MS patients showed a reduction in whole brain and cortical gray matter volumes that was not different among the groups, although the study lacked a group of control subjects [22]. In our study, the mean PBVC/y of MOGAD patients was similar to the value estimated for the general population [23] and to the estimate of PBVC in our control group. Conversely, MS subjects showed a significantly higher PBVC/y compared to controls. Hence, it might be suggested that brain volume loss could be lower, if not absent, in MOGAD patients. A recent study has accordingly shown a significantly lower PBVC/y in NMOSD-AQP4 patients compared to MS patients [24], thus suggesting, together with our findings, that brain atrophy might be related to neuropathological mechanisms that are exclusive to MS. The contrasting evidence of our study compared to others already published could be due to the small sample sizes of each study, thus needing further ascertainment in larger cohorts.

We showed that the T2 lesion load was almost stable over time in MOGAD patients, both in terms of volume and the number of lesions, while it increased in the MS cohort. This finding is in line with a study on MOGAD patients, where MOG-IgG-positive patients initially diagnosed with MS did not show the typical “silent increase” in lesion volume [25]. Moreover, the increase in T2 lesion volume has been proposed as a red flag for the diagnosis of MOGAD [26]. A recent study [27] showed that only 3% of stable MOGAD patients underwent a silent T2 lesion increase, which is in contrast with the robust evidence of the appearance of new asymptomatic lesions in MS patients [28].

Our study suggests that, unlike MS patients, MOGAD subjects are probably not affected by brain atrophy and do not show a significant increase in T2 lesion volume over time. These findings could be related to different neuropathological substrates in the two diseases, with a discrete inflammatory activity in MOGAD patients that, once exhausted, does not switch toward an intrathecal chronic inflammation, which is instead the basis of the neurodegenerative processes in MS [29]. This is in line with the overall better outcome of MOGAD patients, in whom disability—when present—seems only related to attack sequelae and not to the accrual of neurological deficits that occurs in progressive MS. Accordingly, a study on the CSF features of MOGAD cases has shown that the most relevant alterations in terms of blood–brain barrier damage, increased cellularity and CSF proteins are mainly related to the acute attack and tend to recover in the remission phases [30] (p. 1). Moreover, it has been shown that serum neurofilament light chain levels are increased at MOGAD onset, while they tend to decrease over time in most patients [31]. A study on two autoptic cases and twenty-two brain biopsies of MOGAD patients has revealed that demyelinating lesions lack the accumulation of activated microglia at lesion border, which typically occurs in slowly expanding or smoldering white matter MS lesions, thus suggesting the absence of a neuropathological mechanism linked to chronic active inflammation in MS [32].

### 4.2. Brain MRI Lesion Distribution

We showed a clear distinction between MOGAD and MS patients in terms of the distribution, appearance and volume of T2 lesions. In particular, we observed no brain lesions in 50% of MOGAD patients, as previously described by other studies, where up to 50% of MOGAD patients showed normal brain MRI [33,34]. We also confirmed the clear association of cortico-juxtacortical and periventricular lesions with MS, together with the presence of callosal and periventricular signal abnormalities known as Dawson fingers [35]. We observed that MOGAD patients had a significantly lower lesion load compared with the MS cohort and that the brain lesions appeared tumefactive and with blurred margins in MOGAD patients, while in MS subjects, they had an ovoid shape and clearly demarcated borders. These findings are in line with a previous study, which showed that the presence of at least one periventricular/inferior temporal lesion, U-fiber lesion or Dawson finger lesion can differentiate MOGAD from MS with 90% sensitivity and 95% specificity [36].

### 4.3. Cross-Sectional Analysis of Brain Volumes

The mean whole brain volume and WM volume at T0 were significantly higher in MOGAD than in MS patients; conversely, there was no significant difference between HC and MOGAD or MS patients. The role of brain atrophy in MOGAD is still uncertain, and findings from previous studies are discordant. Two studies that compared MOGAD with NMOSD-APQ4 patients and healthy controls did not find a significant difference in whole brain volume between MOGAD cases and controls [37,38]. On the contrary, two other studies showed reduced whole brain volumes in MOGAD subjects compared to controls [22,39]. The reported inconsistencies may be due to the relatively small sample sizes and the different MRI protocols and software used for brain MRI analysis in each of the studies.

There is more consensus regarding a higher WM volume in MOGAD compared to MS patients [22,38,39], as confirmed by our findings. The higher WM volume in MOGAD patients could also be due to a significantly lower T2 lesion volume, with less inflammatory and neurodegenerative effects in this context.

We did not find significant differences in GM volume between MOGAD patients, MS patients and HC subjects. This finding is in line with previous data, where GM volume did not differ between MOGAD patients and HC subjects [37], and with the fact that GM atrophy is known to mainly affect MS patients [9].

### 4.4. Limitations

The main limitation of this study is the absence of a shared MRI protocol across all the enrolled subjects, despite the use of the same scanner for T0 and T1 in each patient and the use of only two scanners for all our cases. However, due to the rarity of MOGAD, we believe that using a multicenter approach to enroll more patients—and to achieve higher statistical power—could in part counterbalance the technical limitations of using different MRI scanners within each participating center.

Another limitation of our study is the comparison of MOGAD and MS patients with a group of HC subjects obtained from an online repository, although matched for sex, age and ethnicity. However, considering the ethical and organizational issues implied by the execution of two MRI exams in healthy people, we believe that the use of online brain MRI repositories might be an acceptable compromise.

Another intrinsic limitation is the different disease duration between the two disease groups (median 8 versus 60 months in MOGAD compared to MS patients), as MOGAD has only recently been considered a specific disease entity. We acknowledge that this difference could in part account for higher brain volume loss in MS patients due to the establishment of neurodegenerative processes that become increasingly relevant during the course of the disease. However, our cohort mainly comprised clinically stable patients with a relapsing-remitting disease course, which is less prone to brain atrophy compared to the progressive phenotypes. A viable turnaround for this limitation could be the inclusion of patients with a disease duration shorter than 10 years in both groups.

## 5. Conclusions

Notwithstanding the limitations, our study corroborates the idea of MOGAD as a distinct entity from MS, with specific pathogenetic mechanisms, as well as clinical and epidemiological features. Further longitudinal prospective studies are needed to confirm and expand our findings.

## Figures and Tables

**Table 1:** Demographic, clinical and radiological features of MOGAD and MS patients.

	MOGAD (*n* = 16)	MS (*n* = 44)	HC (*n* = 14)	*p* Value
Median age at T0—years (range)	36 (21–69)	36 (23–69)	36 (21–71)	0.935
Median age at onset—years (range)	33 (21–68)	31 (16–47)		0.482
Sex—*n* (%)				0.902
F	9 (56.2)	26 (59.1)	9 (64.2)	
M	7 (43.8)	18 (40.9)	5 (35.8)	
Clinical onset—*n* (%)				0.167
Optic neuritis	6 (37.5)	6 (13.6)		
Monolateral	6 (37.5)	5 (11.4)		
Bilateral	0 (0.0)	1 (2.2)		
Myelitis	5 (31.3)	24 (54.5)		
Brainstem	2 (12.5)	9 (20.5)		
Encephalopathy	1 (6.3)	4 (9.1)		
Multifocal	2 (12.4)	1 (2.3)		
MOGAD clinical course—*n* (%)				
Monophasic	11 (68.8)			
Relapsing	5 (31.2)			
Disease duration at T0—median months (range)	8 (0–65)	60 (0–418)		**<0.001**
EDSS—median (range)				
T0	2.0 (0–6)	1.5 (0–4.5)		0.793
T1	0.0 (0–5.5)	1.5 (0–4.0)		0.050
Previous relapses—median (range)	0 (0–3)	1 (0–12)		**0.008**
Relapsed patients during follow-up—*n* (%)	2 (33.3%)	8 (18.2%)		0.384
Interval between MRI scans—months (range)	12 (12–17)	13 (12–18)	24 (24)	0.595
Brain MRI lesions at T0—*n* of patients (%)				
Cortical				**0.007**
0	11 (68.8)	16 (36.3)		
1–2	0 (0.0)	9 (20.5)		
≥3	0 (0.0)	14 (31.8)		
n.a.	5 (31.2)	5 (11.4)		
Juxtacortical				**<0.001**
0	10 (62.4)	5 (11.4)		
1–2	3 (18.8)	4 (9.1)		
≥3	3 (18.8)	35 (79.5)		
Periventricular				**<0.001**
0	8 (50.0)	1 (2.3)		
1–2	6 (37.6)	2 (4.6)		
≥3	2 (12.4)	41 (93.1)		
Thalamus				0.141
0	16 (100.0)	32 (72.7)		
1–2	0 (0.00)	10 (22.7)		
≥3	0 (0.00)	2 (4.6)		
Corpus callosum				**<0.001**
0	16 (100.0)	16 (36.4)		
1–2	0 (0.0)	16 (36.4)		
≥3	0 (0.0)	12 (27.2)		
Basal ganglia				0.415
0	15 (93.8)	35 (79.5)		
1–2	1 (6.2)	9 (20.5)		
≥3	0 (0.0)	0 (0.0)		
Brainstem				**0.001**
0	13 (81.3)	11 (25.0)		
1–2	3 (18.7)	29 (69.9)		
≥3	0 (0.0)	4 (9.1)		
Cerebellar				0.127
0	14 (87.5)	24 (54.5)		
1–2	2 (12.5)	18 (40.9)		
≥3	0 (0.0)	2 (4.6)		
Tumefactive lesions				**0.024**
0	13 (81.2)	43 (97.7)		
≥1	3 (18.8)	1 (2.3)		
Blurred margins				**0.020**
0	12 (75.0)	42 (95.5)		
≥1	4 (25.0)	2 (4.5)		
Contrast enhancement				0.329
0	13 (81.0)	26 (59.2)		
≥1	2 (12.5)	6 (13.5)		
n.a.	1 (6.5)	12 (27.3)		
New T2 lesions				0.178
0	14 (87.5)	31 (70.5)		
≥1	2 (12.5)	13 (29.5)		

## Data Availability

Data are available upon request.

## Abbreviations

The following abbreviations are used in this manuscript:

ADEM	Acute Disseminated Encephalomyelitis
ANOVA	Analysis of Variance
AQP4-Ab	Aquaporin-4 Antibody
CNS	Central Nervous System
CSF	Cerebrospinal Fluid
DIR	Double Inversion Recovery
EDSS	Expanded Disability Status Scale
GM	Gray Matter
HC	Healthy Control
IDE	Initial Demyelinating Event
MOG	Myelin Oligodendrocyte Glycoprotein
MOGAD	Myelin Oligodendrocyte Glycoprotein Antibody-Associated Disease
MRI	Magnetic Resonance Imaging
MS	Multiple Sclerosis
NBV	Normalized Brain Volume
NMOSD-AQP4	Aquaporin-4 Antibody Seropositive Neuromyelitis Optica Spectrum Disorder
ON	Optic Neuritis
PBVC/y	Percentage Brain Volume Change per Year
RRMS	Relapsing-Remitting Multiple Sclerosis
T2LV	T2 Lesion Volume
TM	Transverse Myelitis
WM	White Matter
